# Progress in Surgery

**Published:** 1897-04-17

**Authors:** 


### Progress in Surgery.

### SURGERY OF THE NERVES, &c.

**Nerve Grafting.**—Mr. Mayo Robson[1] has recently reported a case in which, six years ago, he grafted a portion of the spinal cord of a rabbit into the median nerve of a man. The patient had fallen on a scythe cutting the brachial artery, which was ligatured; and dividing a nerve on the lower and inner part of the upper arm, which was sutured. The wound healed by granulation. When seen by Mayo Robson, seven months after the accident, all the muscles acting in the wrist and hand which had their nerve-supply from the median and ulnar nerves were paralysed, while those supplied by the musculo-spiral were capable of acting. Sensation was also considerably interfered with in the distribution of the same nerves. Electrical tests confirmed the opinion that there had been a complete division of the median, ulnar, and internal cutaneous nerves, and that it was useless to attempt anything short of operative interference.

On operating, the ulnar nerve was found embedded in fibrous tissue, the bulbous upper end being connected with the lower by a band of fibrous tissue. This fibrous tissue was excised and a portion of the sciatic nerve of a rabbit used to fill in the gap. The separated ends of the internal cutaneous nerve were united directly by catgut suture. The divided ends of the median nerve were separated by two and a half inches, and a portion of the spinal cord of the rabbit was excised and stitched into the gap with catgut sutures. Healing took place by first intention. Sensation began to reappear in the median area in ten or eleven days, and gradually improved, muscular development and trophic influence following later. Two months after operation he began to have shooting pains along the distribution of the ulnar nerve, and improvement slowly followed. The patient was ultimately able to resume his work as a gardener without any appreciable inconvenience. Robson is not prepared to say whether the segment of the spinal cord took on the functions of a nerve, or simply acted as a basis on which new nerve tissue was built up. It was interesting to observe that the restoration of function followed sooner in the median than in the ulnar nerve, although the interval between the divided ends was much greater in the former. Could it be that the spinal cord formed a better medium for establishing continuity than the sciatic nerve which was used for the ulnar gap? Robson is inclined to doubt the statement made by physiologists that the peripheral portion of a divided nerve always undergoes atrophy.

**Intradural Section of Spinal Nerves for Neuralgia.**—In a paper on the above subject, R. Abbe[2] refers to three cases of his own, and four of other surgeons, where the posterior spinal nerve roots have been divided or resected at their origin from the cord for neuralgias. The writer's opinion is favourable to the operation, which he considers devoid of risk even in weak patients. In all the cases, although the severe paroxysms of pain were relieved at once, the patients continued to suffer twinges at intervals. The operation should be resorted to early in the class of cases of ascending neuritis which have hitherto been treated by successive nerve-stretching and resection, and finally amputation, all without benefit. It has been found from experience that it is necessary to divide two additional roots higher up than the apparent origin of the pain. The fear of dividing the posterior roots of the third and fourth cervical nerves on account of the phrenic appears to be groundless, as this nerve only requires motor supply, and the other phrenic is always intact and in reserve.

**Compression in Traumatic Neuritis.**—In The Hospital of January 4th, 1896, Delorme's[4] paper is referred to. He has added to the list of his cases, which now number ten. The plan suggested is suitable for cases of ascending neuritis following upon injury and associated with severe neuralgic pain, any neurotic element being previously excluded. The method consists in accurately defining the painful area, and then the operator compresses the affected part with all his strength, passing successively all over the hyperæsthetic area. After a few minutes' rest, the compression is repeated if any pain remains. As a rule, one sitting removes the pain permanently; when this does not occur, the procedure may be repeated in a few days. In one case of severe pain in the cicatrix, a bullet wound of the neck, two sittings, at an interval of four days, completely removed the pain, which was still absent three years later. No anæsthetic is employed.

**Resection of the Cervical Sympathetic Nerve in the Treatment of Exophthalmic Goitre and Epilepsy.**—Jonnesco, of Bukarest,[4] has practised the complete bilateral resection of the cervical sympathetic cord in the treatment of various conditions. Several operators have previously exercised more limited portions of the cervical cord and ganglia with varying success. Jonnesco did his first operation in August, 1896, removing the superior and middle ganglia on both sides in a case of exophthalmic goitre. The good results (disappearance of exophthalmos, diminution in size of the thyroid, and lessening of other symptoms) encouraged him to repeat the operation, and even to remove all the ganglia on both sides. He has operated in nine cases—two of exophthalmic goitre, three of genuine epilepsy, one of chorea and hystero-epilepsy, one brain tumour with epileptiform attacks, one hysteria, and one progressive paralysis. At the time of reporting the results were that in both goitre cases the exophthalmos had disappeared, the thyroid diminished; tachycardia had disappeared in one case, but persisted in the other. In the three cases of genuine epilepsy, none of the patients have again had fits, and only one now has occasional slight fits of giddiness. The other cases showed no results. The operation is carried out through an incision along the posterior border of the sterno-mastoid muscle, occupies from a quarter to three-quarters of an hour, and is usually followed by a primary union.

**Surgical Treatment of Spasmodic Wry Neck by Kocher's Method.**—An important contribution on the treatment of spasmodic wry neck is made by F. de Quervain[5] from certain work done in association with Kocher, in Berne. The futility of medical treatment in this condition is admitted by all; and the surgical measures usually practised give but meagre results. Quervain has nothing new to add as regards the etiology or causation of the condition. In the majority of cases either a family or a personal history of predisposition to nervous disturbances is forthcoming, although he has never found spasmodic wry neck as a hysterical manifestation properly so-called. There are three distinct varieties of spasmodic wry neck—the tonic, the clonic, and the tonico-clonic, the first of these being extremely rare. Several varieties of spasm, however, may be met with in the same patient. Quervain draws special attention to the fact that the sterno-clerdo-mastoid muscle is seldom, if ever, the only defaulter, the other neck muscles in nearly every case being also involved in various combinations. The commonest combination is that of the sterno-mastoid of one side with the cervical muscles of the other (8 out of 12 cases). In 3 cases out of 12 the cervical muscles of both sides were involved, and the sterno-mastoid of one side. In the remaining case strong spasm of the left sterno-mastoid and left cervical muscles was associated with slight contraction of the right sterno-mastoid. The association of spasm of sterno-mastoid and cervical muscles on the same side is extremely rare.

In discussing the pathogeny of this condition the author holds that the theories which attribute it to muscular abnormality, and to lesions of the spinal accessory nerve must be abandoned, and a central cause sought for. That this central cause is cortical rather than bulbar he considers almost certain, and he defines spasmodic wry neck as "a functional disturbance of the cortical centre for rotation of the head." If this be admitted, it is evident that medicinal treatment and operative procedures directed against the peripheral nerves must be equally useless. In support of this contention he quotes the result of such operations, sixty-one in number, which showed twelve recoveries, twenty-two improvements, twenty-five failures, and two uncertain results. The logical outcome of his argument is, that treatment should be directed to the diseased spot in the cerebral cortex by trephining and excision of the area. The chance of success by this means, however, is not sufficiently great to counteract the risk of doing more harm than good, besides the dangers incident to the operation. Kocher's method consists in pursuing a purely palliative course by surgical means, dividing every muscle involved in the spasm. In this way the contractions are overcome, without at the same time any collateral disturbances being produced. The first point to determine is the groups of muscles associated in producing the spasms in a given case; and having done this the surgeon then proceeds to perform myotomy of each of these. In the commonest class the sterno-mastoid of one side will be divided through one incision, and the cervical muscles—occipital portion of trapezius, splenius, complexus, and obliquus capitis inferior—of the opposite side, through another. In the other combinations already described appropriate modifications in the operation will be made. The twelve cases operated upon by Kocher show the following results: In seven recovery took place, the spasm disappeared, the head was in good position, and nothing inconvenient remained beyond a feeling of rigidity or weight in the cervical region. These results have lasted for periods varying from six months to 10 and 12 years. Three patients were benefited, but not cured by the operation. In all of them Kocher is desirous of repeating or supplementing the myotomies, believing that benefit would still result. The two remaining cases are still under treatment, but as yet show no improvement. No serious complaints are made by patients as to inconvenience in the movement or position of the head after this operation.

Quervain would explain the good results obtained on one of two theories—either the muscles never unite sufficiently to enable them to compete successfully with their antagonists; or the myotomy exerts an indirect influence on the cortical centre.

**Treatment of Spasmodic Lockjaw.**—By an operation on somewhat similar lines to that for spasmodic wry neck, Kocher[6] treats cases of spasmodic lockjaw where no gross cause can be discovered for the condition. He reports a case of a woman, aged 28, who, some years before coming to him, began to suffer from firm closure of the jaws, varying in degree from day to day, but gradually getting worse. The tempero-maxillary joint and a displaced cartilage were excised, with benefit lasting only for two or three days; and other methods of treatment were equally unsatisfactory. At last, after one of the insertions of the masseter, temporal and internal pterygoid were detached, and the spasms entirely disappeared and remained absent for six years. They then recurred, and a similar operation was carried out.

[1] B.M.J., Oct. 31, 1896. [2] Boston Med. and Surg. Journ., Oct. 1, 1896. [3] Journ. de Méd., June, 1896. [4] Central für Chirurg, Jan. 16, 1897. [5] Med. Week, 1896, p. 565. [6] Sém. Méd., Dec. 18, 1896.

